# Unraveling the modulatory manner and function of circRNAs in the Asian honey bee larval guts

**DOI:** 10.3389/fcell.2024.1391717

**Published:** 2024-07-09

**Authors:** Xuze Gao, He Zang, Xiaoyu Liu, Sijia Guo, Daoyou Ye, Zhitan Liu, Xin Jing, Qingsheng Niu, Ying Wu, Yang Lü, Dafu Chen, Rui Guo

**Affiliations:** ^1^ College of Bee Science and Biomedicine, Fujian Agriculture and Forestry University, Fuzhou, China; ^2^ National and Local United Engineering Laboratory of Natural Biotoxin, Fuzhou, China; ^3^ Apitherapy Research Institute of Fujian Province, Fuzhou, China; ^4^ Apiculture Science Institute of Jilin Province, Jilin, China; ^5^ Mudanjiang Branch of Heilongjiang Academy of Agricultural Sciences, Mudanjiang, China

**Keywords:** non-coding RNA, circRNA, honey bee, *Apis cerana*, gut, development, regulatory mechanism

## Abstract

Circular RNAs (circRNAs) are a class of non-coding RNAs (ncRNAs) that can participate in biological processes such as gene expression, growth, and development. However, little has been explored about the function of circRNAs in the development of *Apis cerana* larval guts. By using our previously gained deep sequencing data from the guts of *A. cerana* worker larvae at 4-, 5-, and 6-day-old (Ac4, Ac5, and Ac6 groups), the expression pattern and regulatory role of circular RNAs (circRNAs) during the development process was comprehensively investigated, with a focus on differentially expressed circRNAs (DEcircRNAs) relevant to immunity pathways and developmental signaling pathways, followed by validation of the binding relationships among a key competing endogenous RNA (ceRNA) axis. Here, 224 (158) DEcircRNAs were detected in the Ac4 vs. Ac5 (Ac5 vs. Ac6) comparison group. It’s suggested that 172 (123) parental genes of DEcircRNAs were involved in 26 (20) GO terms such as developmental process and metabolic process and 138 (136) KEGG pathways like Hippo and Wnt signaling pathways. Additionally, ceRNA network analysis indicated that 21 (11) DEcircRNAs could target seven (three) DEmiRNAs, further targeting 324 (198) DEmRNAs. These DEmRNAs can be annotated to 33 (26) GO terms and 168 (200) KEGG pathways, including 12 (16) cellular and humoral immune pathways (endocytosis, lysosome, Jak-STAT, etc.) and 10 (nine) developmental signaling pathways (Hippo, mTOR, Hedgehog, etc.). Interestingly, DEcircRNAs in these two comparison groups could target the same ace-miR-6001-y, forming complex sub-networks. The results of PCR and Sanger sequencing confirmed the back-splicing sites within four randomly selected DEcircRNAs. RT-qPCR detection of these four DEcircRNAs verified the reliability of the used transcriptome data. The results of dual-luciferase reporter assay verified the binding relationships between novel_circ_001627 and ace-miR-6001-y and between ace-miR-6001-y and *apterous-like*. Our data demonstrated that DEcircRNAs were likely to modulate the developmental process of the *A. cerana* worker larval guts via regulation of parental gene transcription and ceRNA network, and novel_circ_001627/ace-miR-6001-y/*apterous-like* was a potential regulatory axis in the larval gut development. Findings from this work offer a basis and a candidate ceRNA axis for illustrating the circRNA-modulated mechanisms underlying the *A. cerana* larval guts.

## 1 Introduction

Honey bee is one of the most important pollinating insects in nature and plays an essential part in ecological balance and food security (Tan et al., 2022). *Apis cerana* is a major bee species widely reared in many parts of Asian countries, with a subseries of advantages like adaptation to diverse environments, ability of fast dispersal, ecological balance maintenance and crop pollination (Gallai et al., 2009). The gut of insects including honeybee is a major tissue responsible for food digestion, nutrient absorption, and immune defense (Holtof et al., 2019; Khan et al., 2023). However, the developmental mechanisms underlying the honeybee gut is currently still largely unknown.

Non-coding RNAs (ncRNAs), such as miRNA, lncRNA, and circular RNA (circRNA), represent a layer of regulators in various biological processes (Zhang et al., 2019). NcRNAs can be divided into housekeeping ncRNAs and regulatory ncRNAs, and the latter is usually considered to play a critical part in controlling gene expression at transcriptional or post-transcriptional level (Leng et al., 2022). As a new member of regulatory ncRNA family, circRNAs are generated by the back-splicing of pre-mRNAs (Li et al., 2018b). Since circRNAs have special covalently closed-loop structure that lacks 5′cap and 3′ployA tail, circRNAs are more resistant to RNase R enzyme digestion than linear RNAs and therefore regarded as ideal endogenous biomarker (Meng et al., 2017; Kristensen et al., 2019).

Abundant circRNAs have been discovered in animals, plants, and microorganisms, such as *H. sapiens* (Liu et al., 2019), *Triticum aestivum* (Xu et al., 2019) and *Varroa destructor* (Lin et al., 2021). Accumulating evidence have shown that circRNAs played vital roles in diverse processes, such as development, metabolism, and immunity (Liu et al., 2019; Zhou et al., 2020; Shen et al., 2024). Recent studies on human diseases suggested that circRNAs can serve as novel biomarkers, therapeutic agents, and drug targets (Xin et al., 2021; Kristensen et al., 2022). Compared to mammals like *Homo sapiens*, study regarding circRNAs in insects is lagging. Limited advancements have gained in a few model insects such as *Drosophila* (Weigelt et al., 2020), *Aedes albopictus* (Gao et al., 2023), and *Bombyx mori* (Wang et al., 2020). For instance, Gao et al. (2023) demonstrated that circRNA-407 acted as a sponge of aal-miR-9a-5p to promote the expression of the target gene *Foxl* and eventually modulated ovarian development. In recent years, the findings from other groups and our team suggested that circRNAs were potential regulators in western honey bee (*Apis mellifera*) ovary activation and oviposition (Chen et al., 2019a; Chen et al., 2023 X.), brain nerve apoptosis (Shan et al., 2023), task allocation (Thölken et al., 2019), and midgut growth and development (Guo et al., 2018). Chen X. et al. (2023) detected that overexpression of ame_circ_0002015 enhanced the number of eggs laid by *A. mellifera* queens, and further verified that ame_circ_0002015 was capable of sponging ame_miR-14-3p.

Current understanding of *A. cerana* circRNAs is even more scarce. Chen et al. (2020) previously identified 9,589 circRNAs in the midgut tissues of *A. cerana* workers and revealed that differentially expressed circRNAs (DEcircRNAs) between 8- and 11-day-old group were likely to participate in the developmental process of midgut through diverse manners such as competing endogenous RNA (ceRNA). By deciphering the circRNA responses of *A. cerana* to infections by *Ascosphaera apis* and *N*. *ceranae*, our group observed that the expression pattern of host circRNAs was altered by these two widespread fungal bee pathogens and DEcircRNAs were involved in host responses including immune response (Zhu et al., 2022; Guo et al., 2024). However, it is still unknown whether and how circRNAs regulate the growth and development of the larval guts of *A. cerana*. Therefore, it is necessary and meaningful to investigate the regulatory manners and functions of circRNAs in the development of *A. cerana* larval guts.

In the present study, based on our previously obtained high-quality transcriptome datasets, we investigated the differential expression profile of circRNAs during the developmental process of *A. cerana* worker larval gut, followed by dissection of the regulatory manner and role of DEcircRNAs. Additionally, we analyzed the DEcircRNA-engaged sub-networks associated with immune pathways as well as developmental signaling pathways. Furthermore, the binding relationships between key DEcircRNA and target DEmiRNA as well as DEmiRNA and target DEmRNA were verified by molecular method. Findings from this current work offer a novel insight into the epigenetic regulation of the development of *A. cerana* worker larval gut, provide candidate DEcircRNA/DEmiRNA/DEmRNA axis for elucidating the mechanism underlying gut development.

## 2 Materials and methods

### 2.1 Bee larvae

*A. cerana* worker larvae were obtained from three colonies reared in the teaching apiary of College of Bee Science and Biomedicine at Fujian Agriculture and Forestry University (119.2369° E, 26.08279° N), Fuzhou city, Fujian province, China.

### 2.2 RNA-seq data source

In our previous study, the 4-, 5-, and 6-day-old gut tissues of *A. cerana* worker larvae were prepared, and respectively named Ac4 (Ac4-1, Ac4-2, and Ac4-3 were three biological replicates), Ac5 (Ac5-1, Ac5-2, and Ac5-3 were three biological replicates), and Ac6 (Ac5-1, Ac5-2, and Ac5-3 were three biological replicates), followed by RNA isolation, strand-specific cDNA library construction, RNA sequencing, quality control of raw reads, and genomic mapping of clean reads, which included circRNA (Guo et al., 2024) and mRNA data (Guo et al., 2019). The raw data were deposited in the NCBI Sequence Read Archive (SRA) database (www.ncbi.nlm.nih.gov) and linked to the BioProject number: PRJNA560730 and SRA456721.

### 2.3 sRNA-seq data source

In another previous work, the aforementioned gut tissues of *A. cerana* worker 4-, 5-, and 6-day-old larvae were subjected to total RNA isolation, cDNA library construction, sRNA-seq, and data quality control (Fan et al., 2023). The raw data were available in the NCBI SRA database under the BioProject number: PRJNA565611.

### 2.4 Expression calculation and differential analysis of circRNAs

The expression level of each circRNA was calculated based on the RPM (mapped back-splicing junction reads per million mapped reads) method (Zhang et al., 2014) with the formula: RPM = 10^6^C/N, where RPM is the expression level of a circRNA, C is the number of back spliced reads aligned to this circRNA, N is the total number of back spliced reads aligned to all circRNAs in the *A. cerana* reference genome (assembly ACSNU-2.0). Next, according to the method: (circRNA’s RPM in Ac5)/(circRNA’s RPM in Ac4) as well as (circRNA’s RPM in Ac6)/(circRNA’s RPM in Ac5), the Fold Change (FC) between Ac4 vs. Ac5 and Ac5 vs. Ac6 comparison groups was computed. Differential expression analysis was then performed, DEcircRNAs in the two comparison groups following the criteria of |log_2_FC| > 1 and *p* < 0.05 (corrected by the false discovery rate); *p* < 0.05, log_2_FC > 1 as upregulated DEcircRNAs and *p* < 0.05, log_2_FC < 1 as downregulated DEcircRNAs. Venn analysis and expression clustering of shared DEcircRNAs in the two comparison groups were performed by using relevant tools in the OmicShare platform (https://www.omicshare.com/). The RPM values of shared DEcircRNAs were uploaded to the tool, and the RPM value of each DEcircRNA in three groups was normalized using the Z-score method.

### 2.5 Prediction and investigation of parental genes

The anchor reads at both ends of DEcircRNAs were mapped to the *A. cerana* reference genome (assembly ACSNU-2.0) using the Bowtie2 v2.3.4.2 software (Langmead and Salzberg, 2012), if both ends of a DEcircRNA were aligned with the same gene, the gene was considered to be the parental gene of this DEcircRNA. To gain functional and pathway annotations, the parental genes of corresponding DEcircRNAs were mapped to the GO (http://www.geneontology.org/) and KEGG (https://www.kegg.jp) databases. Next, circle diagrams and chord diagrams were drawn in the OmicShare platform.

### 2.6 Construction and analysis of ceRNA networks and sub-networks

The target binding relationships of DEcircRNA-targeted DEmiRNAs and DEmiRNA-targeted DEmRNAs were respectively predicted by using a collaboration of three software including miRanda (v3.3a) (Ritchie, 2017), RNAhybrid (v2.1.2) + svm_light (v6.01) (Rehmsmeier et al., 2004; Krüger and Rehmsmeier, 2006), and TargetFind software (Allen et al., 2005). The intersection of the predicted results was regarded as the final targets with high credibility. Further, the DEcircRNA/DEmiRNA/DEmRNA regulatory networks were constructed on basis of the targeting relationships, followed by visualization with the Cytoscape v.3.2.1 software (Smoot et al., 2011). GO term and KEGG pathway analyses of the above DEmRNAs were the same as those described in Section 2.5.

### 2.7 PCR validation and sanger sequencing of DEcircRNAs

Four DEcircRNAs, including novel_circ_002852, novel_circ_002548, novel_circ_002848 and novel_circ_000291, were randomly chosen for PCR amplification and Sanger sequencing. The divergent primers for each DEcircRNA were designed with Primer Premier six software (Supplementary Table S1) and synthesized by Sangon Biotech Co., Ltd. (Shanghai, China). Then, the total RNA from the larval gut samples in the Ac4, Ac5, and Ac6 groups were respectively isolated by the TaKaRa MiniBEST Universal RNA Extraction Kit (TaKaRa, Japan) and digestion of linear RNA with 3 U/mg RNase R to enrich circRNAs. After 37 °C for 15 min, used random primers for reverse transcription and obtained the corresponding cDNA. These cDNA were then used for PCR amplification with the system: cDNA, 1 μL; PCR Mix, 10 μL; upstream primers and downstream primers (2.5 pmol/L), 1 μL, respectively; and sterile water, 7 μL. The reaction was under the following conditions: 95°C for 5 min, 95°C for 30 s, and 60°C for 30 s, for 34 cycles; then, 72°C for 2 min. Next, checked the amplified products through 1.5% agarose gel electrophoresis with GoldView staining (Accurate, China), and the target fragments was purificated with the FastPure Gel DNA Extraction Mini Kit (Vazyme, China). The purificated target fragments was then ligated into the pESI-T vector (Yeasen, China), and inverted into *Escherichia coli* DH5*α* competent cells. Subsequently, Sanger sequencing was sent to Sangon Biotech Co., Ltd. (Shanghai, China).

### 2.8 Stem-loop RT-PCR validation and sanger sequencing of ace-miR-6001-y

To verify the authenticity of miRNA, forward primers (F), universal reverse primers (R) and specific stem-loop primers for ace-miR-6001-y were designed and synthesized (Supplementary Table S2). The total RNA of 5-day-old larvae gut tissues (n = 9) was extracted followed by reverse transcription with stem-loop primers to gain cDNA templates. Following PCR amplification, 1.5% agarose gel electrophoresis and Sanger sequencing were conducted.

### 2.9 RT-qPCR detection of DEcircRNAs

The above-mentioned four DEcircRNAs were subjected to RT-qPCR detection. The *actin* gene (GenBank accession number: 107999330) was used as an internal reference. Following isolating the total RNA from 4-, 5-, and 6-day-old larvae gut tissues (n = 9), respectively, the first-strand cDNAs were synthesized with corresponding divergent primers via the Hifair^®^ III first Strand cDNA Synthesis Kit (gDNA digester plus) (Yeasen, China), and then, the resulting cDNA was used as templates for the qPCR reaction on a ABI QuantStudio 3 Real-Time PCR System (Thermofisher, USA), following the conditions: pre-denaturation under 95 °C for 5 min, denaturation under 95 °C for 15 s, extension under 60 °C for 30 s, and a total of 40 cycles. This experiment was repeated three times. Used the 2^−ΔΔCt^ method to calculate the above DEcircRNAs’ relative expression level (Livak and Schmittgen, 2001). Based on the method described by Liu et al. (2021), the data were presented as the mean ± standard deviation (SD) and analyzed with two-sided Student’s t-test utilizing the Graph Prism eight software (ns *p* > 0.05; *p < 0.05; **p < 0.01; ***p < 0.001).

### 2.10 Dual-luciferase assay

Due to the frequent occurrence of ace-miR-6001-y in regulatory network analysis, novel_circ_001627 and ncbi_107995149 (*apterous-like*) in the regulatory network axis were randomly selected to verify the target binding relationship with ace-miR-6001-y. Following the method of Lan et al. (2023), the specific binding sites of novel_circ_001627 and ace-miR-6001-y as well as between *apterous-like* 3′-UTR and ace-miR-6001-y were respectively forecasted using the RNA hybrid software. Specific primers for the above binding sequences (Supplementary Table S3) were synthesized by Sangon Biotech Co., Ltd. (Shanghai, China) and cloned into pmirGLO vectors (Promega, China), generating the recombinant plasmids named pmirGLO-circ001627-wt and pmirGLO-mRNA5149-wt. Recombinant plasmids of the corresponding mutant sequences (Supplementary Table S3) were synthesized with the same method, and named pmirGLO-circ001627-mut and pmirGLO-mRNA5149-mut. Next, ace-miR-6001-y mimic (mimic-ace-6001-y) and negative control mimic (mimic-negative control, mimic-NC) were designed and synthesized by GenePharma (Shanghai, China) Co., Ltd. Then, extracted plasmids through the EasyPure^®^ HiPure Plasmid MiniPrep Kit (Transgenbiotech, China).

Next, thawed the cryogenic storage tube containing 1 mL of HEK-293T cell suspension by shaking rapidly in a 37 °C water bath and made the cell resuspension, then added it to a cell culture flask containing 6–8 mL of complete medium (Dulbecco’s Modified Eagle Medium: 89%, Fetal Bovine Serum: 10%, Penicillin-Streptomycin Solution: 1%) and placed into a 37 °C incubator for 24 h. Passage was performed when the cell density reaches 70%–90%. Next, the HEK-293T cells were spread into 96-well cell culture plate and then placed into the 37 °C incubator for 24 h to reach a cell density of 90%–95%. Then, followed by co-transfection of mimic-ace-6001-y and pmirGLO-circ001627-wt/mut as well as mimic-NC and pmirGLO-circ001627-wt/mut into the HEK-293T cells using the Hieff Trans^®^ Liposomal Transfection Reagent (Yeasen, China), and the same treatment was performed for pmirGLO-mRNA5149-wt/mut. By using a dual-luciferase detection kit (Yeasen, China), the viability of firefly fluoresceinase and Renilla fluoresceinase was detected on a dual-luciferase assay reporter system (Promega, USA). This experiment was repeated in triplicate. On basis of the method of Matsumura et al. (2022) and Wang et al. (2023), the data were shown as the means ± standard deviation (SD) and were subjected to two-sided Student’s t-test by the Graph Prism eight software (ns, *p* > 0.05; *, *p* < 0.05; **, *p* < 0.01; ***, *p* < 0.001).

## 3 Results

### 3.1 Differential expression pattern of circRNAs during the developmental process of larval guts

**FIGURE 1:**
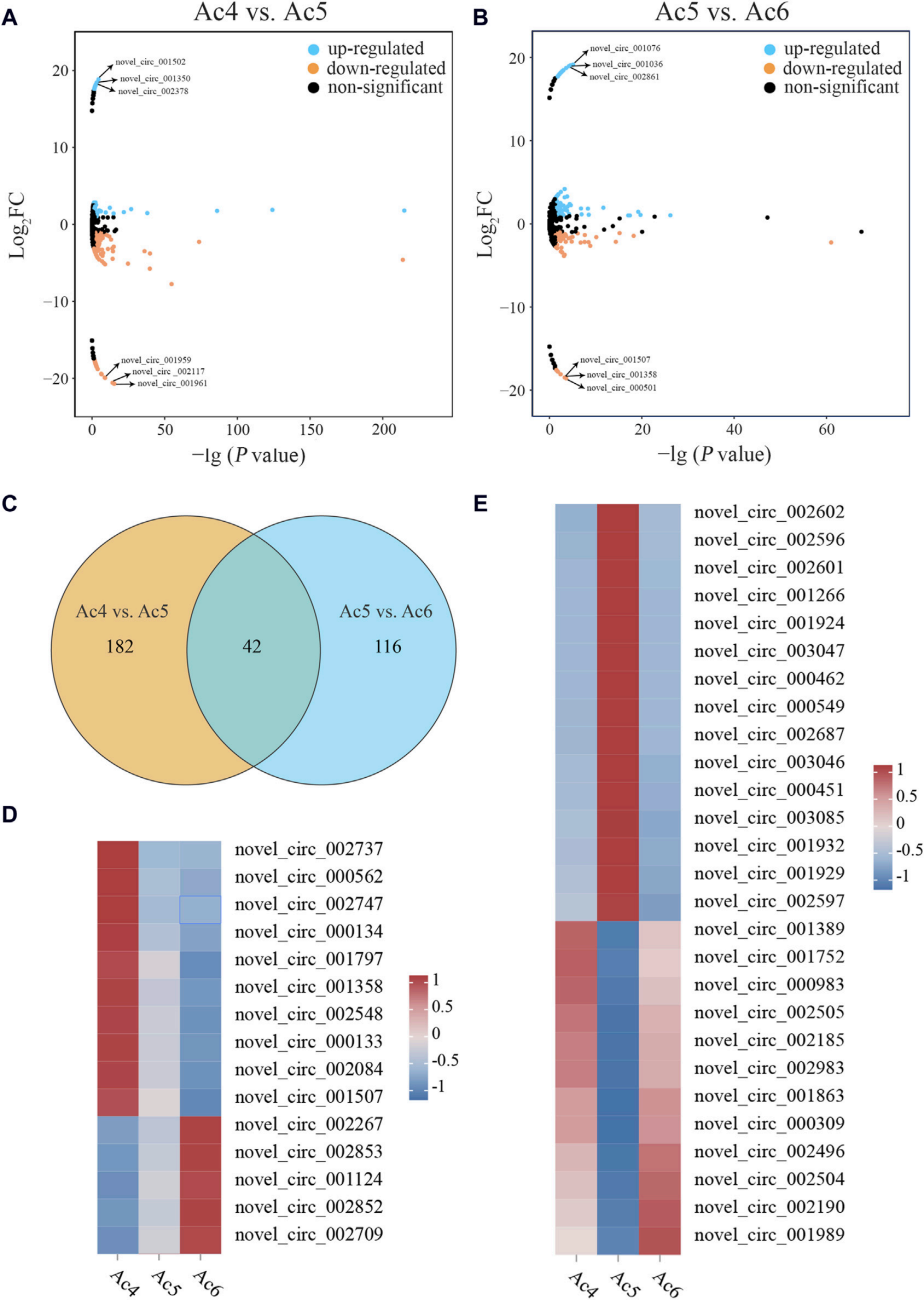
Differential analysis of circRNAs during the developmental process of the *A. cerana* worker larval guts. **(A, B)**: Manhattan maps of DEcircRNAs and non-significant circRNAs in the Ac4 vs. Ac5 and Ac5 vs. Ac6 comparison groups. The blue points indicate the upregulated DEcircRNAs, the orange points indicate the downregulated DEcircRNAs, and the black points indicate the non-significant circRNAs. The black arrows indicate the three most up- and downregulated DEcircRNAs in the two comparison groups. **(C)**: Venn diagram of DEcircRNAs in the two comparison groups. The intersection represents the shared ones. **(D)**: Heatmap of expression clustering for the shared DEcircRNAs showing continuous expression trends. **(E)**: Heatmap of expression clustering for the shared DEcircRNAs presenting various expression trends.

In the Ac4 vs. Ac5 comparison group, 224 DEcircRNAs were detetcted, including 55 up- and 169 downregulated ones, and 2,954 non-significant circRNAs were meanwhile screened (Figure 1A). Among these DEcircRNAs, the three most upregulated ones were novel_circ_001502 (log_2_FC = 18.85, *p* = 3 × 10^−5^), novel_circ_001350 (log_2_FC = 18.47, *p* = 0.0004), and novel_circ_002378 (log_2_FC = 18.23, *p* = 0.001), while the three most downregulated ones were novel_circ_001961 (log_2_FC = −20.71, *p* = 4 × 10^−16^), novel_circ _002117 (log_2_FC = −20.68, *p* = 9 × 10^−16^), and novel_circ_001959 (log_2_FC = −20.59, *p* = 7 × 10^−15^). Comparatively, 158 DEcircRNAs (91 up- and 67 downregulated ones) as well as 3,020 non-significant circRNAs were observed in the Ac5 vs. Ac6 comparison group (Figure 1B). Among these DEcircRNAs, the most upregulated one was novel_circ_001076 (log_2_FC = 19.15, *p* = 7 × 10^−6^) followed by novel_circ_001036 (log_2_FC = 19.05, *p* = 3 × 10^−5^) and novel_circ_002861 (log_2_FC = 18.95, *p* = 6 × 10^−5^), whereas the most downregulated one was novel_circ_000501 (log_2_FC = −18.57, *p* = 0.0002) followed by novel_circ_001358 (log_2_FC = −18.47, *p* = 0.0004) and novel_circ_001507 (log_2_FC = −18.47, *p* = 0.0004). In addition, 42 DEcircRNAs were shared by these two comparison groups (Figure 1C). Following expression clustering analysis, it’s found that 15 DEcircRNAs displayed continous expression trends with the developmental time, including continuously increase trend (novel_circ_002267, novel_circ_002852, and novel_circ_001124, etc.) and continuously decrease trend (novel_circ_002737, novel_circ_001797, and novel_circ_002747, etc.) (Figure 1D), while other 27 DEcircRNAs presented various expression trends, including increase-decrease trend (novel_circ_002602, novel_circ_001924, and novel_circ_002687, etc.) and decrease-increase trend (novel_circ_001752, novel_circ_002505, and novel_circ_001989, etc.) (Figure 1E). Detailed information about the circRNAs were shown in Supplementary Table S4.

### 3.2 Investigation of parental genes of DEcircRNAs

**FIGURE 2:**
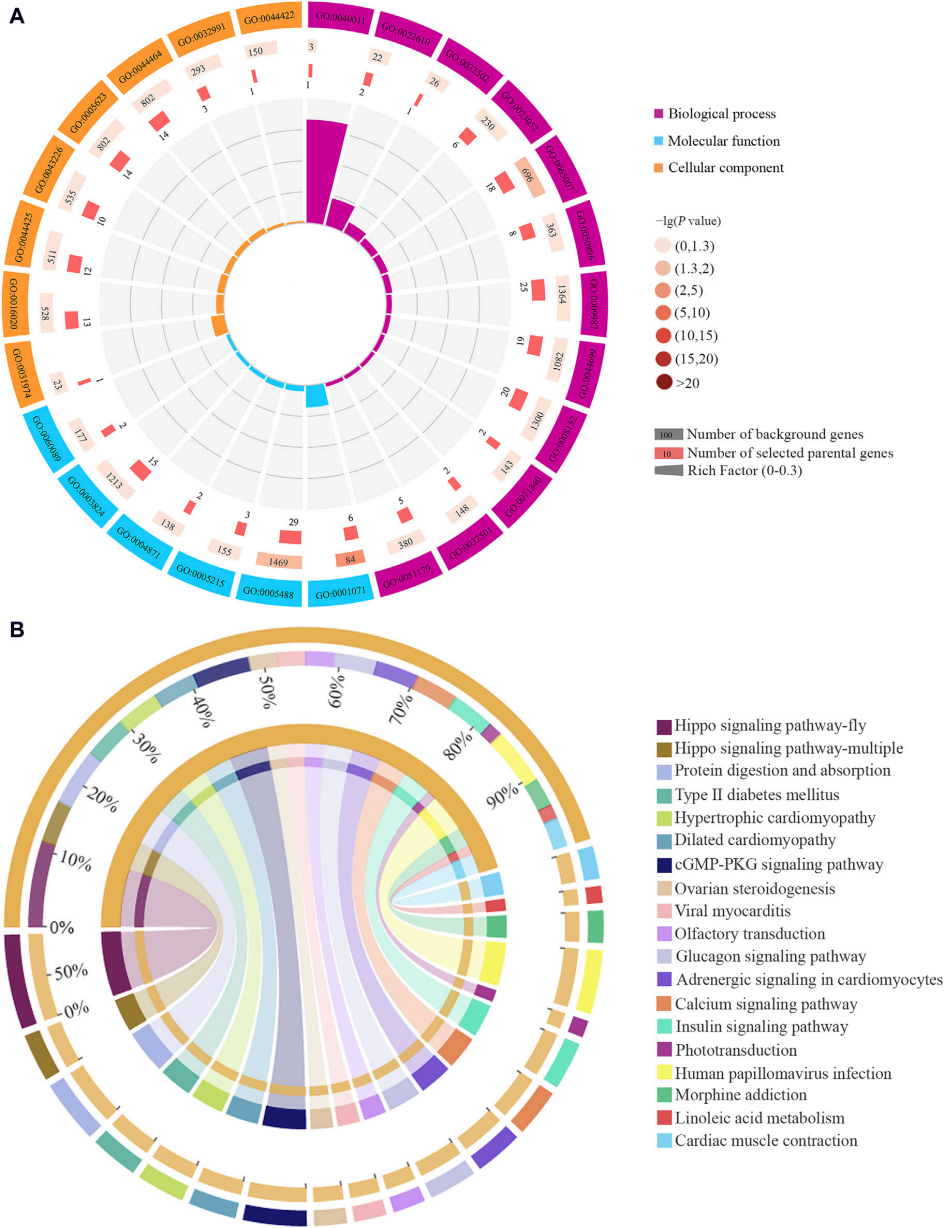
Functional terms and pathways annotated by parental genes of DEcircRNAs in the Ac4 vs. Ac5 comparison group. **(A)**: Loop graph of GO terms annotated by parental genes. **(B)**: Circos graph of KEGG pathways enriched by parental genes. The scale value represents the proportion of the corresponding color label.

CircRNAs have been confirmed to be able to regulate the transcription of their parental genes (Shao et al., 2020). In the Ac4 vs. Ac5 comparison group, 224 DEcircRNAs were predicted to regulate 172 parental genes, involving 26 GO terms related to biological process, molecular function, and cellular component, such as biological regulation, transporter activity, and cell (Figure 2A, see also Supplementary Table S5). These parental genes were also engaged in 138 KEGG pathways, namely, PI3K-Akt signaling pathway, protein digestion and absorption, and Insulin signaling pathway (Figure 2B, see also Supplementary Table S6). Further analysis showed that nine parental genes were associated with eight immune pathways such as lysosome and MAPK signaling pathway (Table 1), 12 ones were relative to nine developmental signaling pathways such as Hedgehog and AMPK (Table 1), and seven ones were relevant to 11 material metabolism-associated pathways such as linoleic acid metabolism (Table 1).

**TABLE 1:** Details of parental genes associated with immune pathways, developmental signaling pathways, and material metabolism pathways.

	Pathway	Gene number	*p*-value
Developmental signaling pathways	Hippo signaling pathway-fly	6	0.0001376885
	Hippo signaling pathway-multiple species	3	0.003429118
	AMPK signaling pathway	2	0.240983
	Hedgehog signaling pathway	1	0.2708857
	Hedgehog signaling pathway-fly	1	0.311744
	Hippo signaling pathway	1	0.6367544
	Wnt signaling pathway	1	0.6523531
	mTOR signaling pathway	1	0.7168958
	Insulin signaling pathway	3	0.07603756
Immune pathways	MAPK signaling pathway	3	0.15797
	MAPK signaling pathway-fly	1	0.6862447
	Ras signaling pathway	2	0.3259448
	cAMP signaling pathway	3	0.1511565
	Lysosome	1	0.6672956
	Endocytosis	2	0.5185137
	Phagosome	1	0.5608659
	Melanogenesis	1	0.534539
Material metabolism-related pathways	Linoleic acid metabolism	1	0.1210132
	Arachidonic acid metabolism	1	0.216463
	Ether lipid metabolism	1	0.2386932
	Glycerolipid metabolism	1	0.4378562
	Glycerophospholipid metabolism	1	0.5917319
	Starch and sucrose metabolism	1	0.2495756
	Pyruvate metabolism	1	0.4213338
	Amino sugar and nucleotide sugar metabolism	1	0.4617775
	Glycolysis/Gluconeogenesis	1	0.5277196
	Arginine biosynthesis	1	0.1580707
	Alanine, aspartate and glutamate metabolism	1	0.3216032

Comparatively, 158 DEcircRNAs in the Ac5 vs. Ac6 comparison group potentially modulated 123 parental genes, involving a total of 20 functional terms such as cellular process, binding, and organelle (Supplementary Figure S1, see also Supplementary Table S7). Additionally, these parental genes were involved in 136 pathways such as apoptosis-fly, aldosterone synthesis and secretion, and MAPK signaling pathway (Supplementary Figure S1, see also Supplementary Table S8). Further, it was found that 10 parental genes were enriched in nine immune pathways (melanogenesis, cAMP signaling pathways, apoptosis, etc.), five parental genes were enriched in seven developmental signaling pathways (Hippo, Wnt, Hedgehog ect.), and five parental genes were enriched in seven material metabolism-relevant pathways (arginine biosynthesis, ether lipid metabolism, inositol phosphate metabolism, etc.), as shown in Supplementary Figure S1.

### 3.3 DEcircRNA-engaged ceRNA networks in the larval guts

**FIGURE 3:**
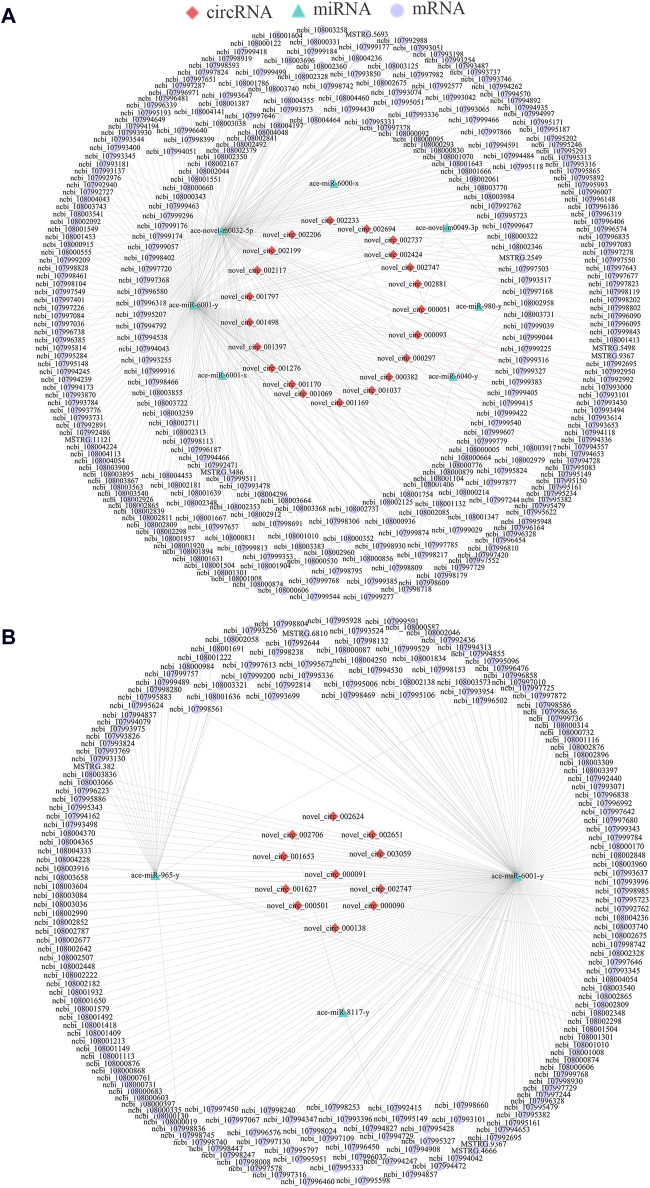
DEcircRNA-DEmiRNA-DEmRNA networks in the *A. cerana* worker larval guts. **(A)**: CeRNA network in Ac4 vs. Ac5 comparison group. **(B)**: CeRNA network in Ac5 vs. Ac6 comparison group.

As a kind of versatile regulators, circRNAs could also act as “molecular sponges” to absorb target miRNAs, further affecting downstream gene epxression and relevant biological processes (Chen et al., 2019b). Here, 21 DEcircRNAs in the Ac4 vs. Ac5 comparison group were detected to target seven DEmiRNAs and further target 324 DEmRNAs (Figure 3A), involving a total of 33 GO terms (biological regulation, cell, binding, etc.) (Supplementary Table S9) and 168 KEGG pathways (Hippo, Wnt, and Oxytocin signaling pathway, ect.) (Supplementary Table S10). In contrast, 11 DEcircRNAs in the Ac5 vs. Ac6 comparison group were observed to target three DEmiRNAs and further target 198 DEmRNAs (Figure 3B), involving 26 functional terms (biological adhesion, catalytic activity, membrane, etc.) (Supplementary Table S11) and 200 pathways (RNA polymerase, melanogenesis, lysosome, etc.) (Supplementary Table S12).

### 3.4 Immunity-associated sub-networks in the larval guts

**FIGURE 4:**
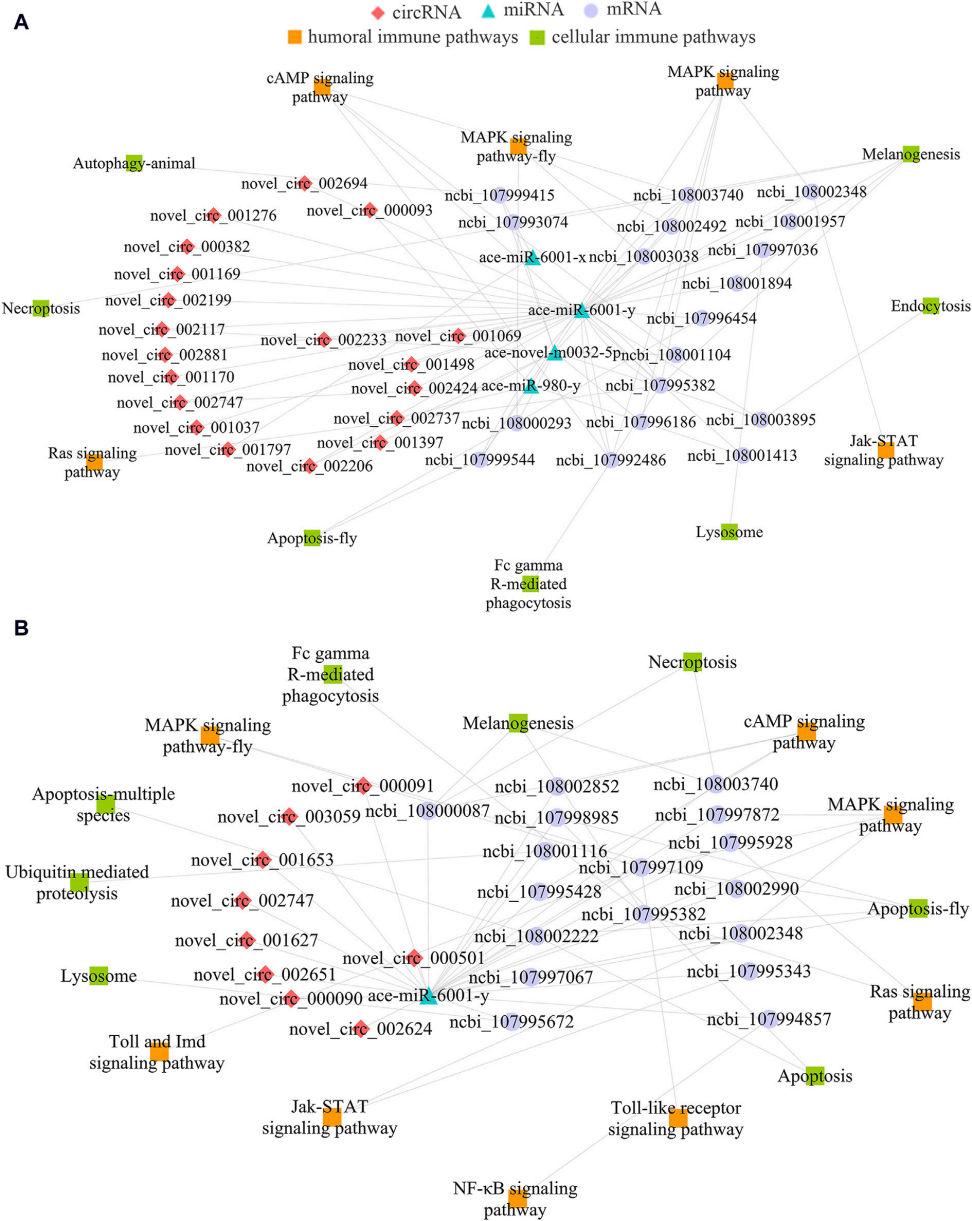
DEcircRNA-involved sub-networks relevant to cellular and humoral immune pathways. **(A)**: Immune-related sub-network in Ac4 vs. Ac5 comparison group. **(B)**: Immune-related sub-network in Ac5 vs. Ac6 comparison group.

Considering honeybee gut is a pivotal immune tissue, sub-networks associated with cellular and humoral immune were further investigated, it’s found that 19 DEcircRNAs in the Ac4 vs. Ac5 comparison group could target four DEmiRNAs and further target 18 DEmRNAs, relative to seven cellular immune pathways (necroptosis, melanogenesis, lysosome, etc.) and five humoral pathways (MAPK, cAMP, Jak-STAT signaling pathway, etc.) (Figure 4A). Additionally, nine DEcircRNAs in the Ac5 vs. Ac6 comparison group could target only one DEmiRNA (ace-miR-6001-y), further targeting 17 DEmRNAs associated with eight cellular immune pathways (Fc gamma R-mediated phagocytosis, necroptosis, apoptosis, etc.) and eight humoral immune pathways (Toll and Imd, Ras, and NF-*κ*B signaling pathway, etc.) (Figure 4B).

### 3.5 Developmental signaling pathway-relevant sub-networks

**FIGURE 5:**
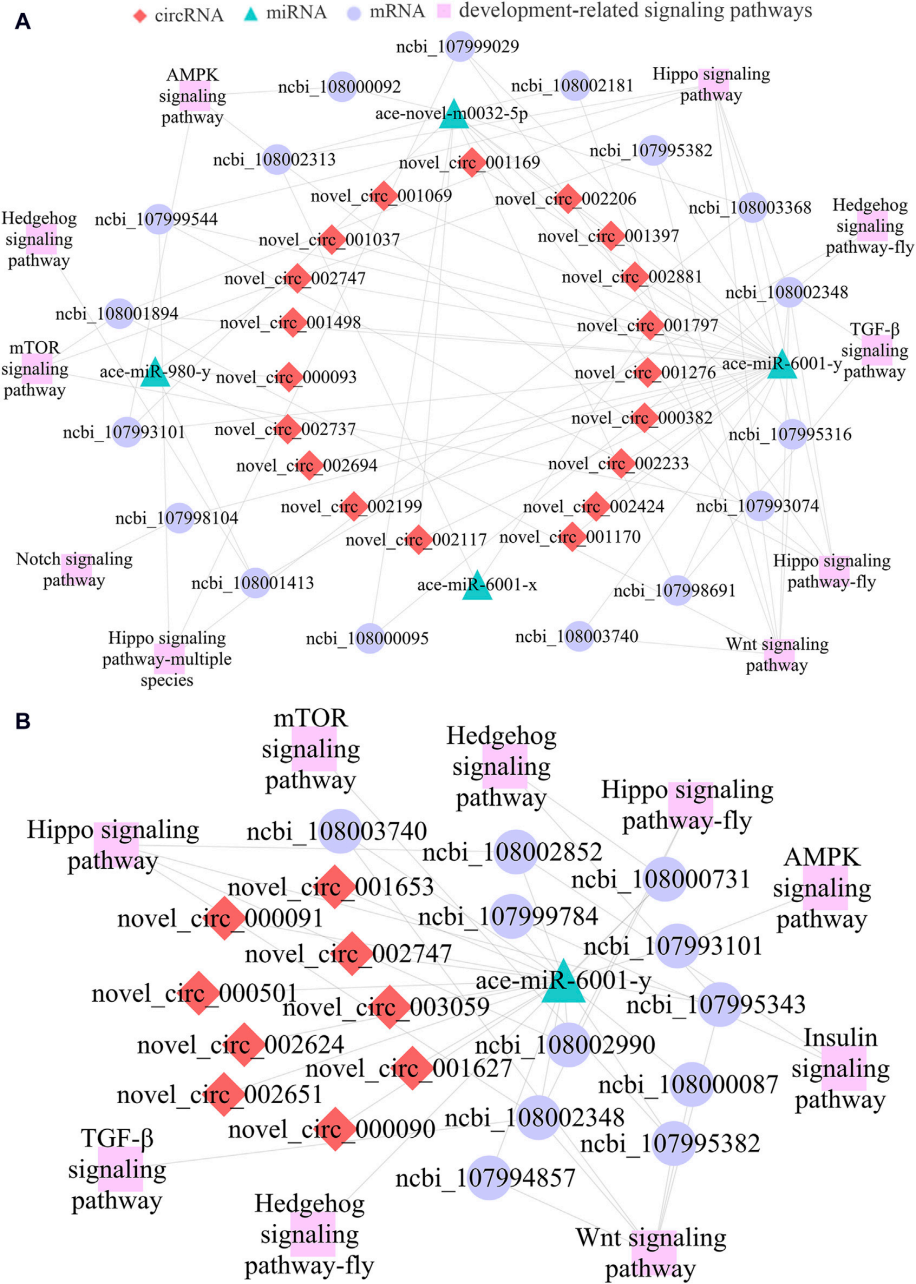
CeRNA regulatory networks of DEcircRNAs relevant to development-associated signaling pathways. **(A)**: Development-related sub-network in Ac4 vs. Ac5 comparison group. **(B)**: Development-related sub-network in Ac5 vs. Ac6 comparison group.

In view of that signaling pathways were closely related to the growth and development of honeybee gut, sub-networks relative to several crucial developmental signaling pathways were further analyzed, the results indicated that 19 DEcircRNAs in the Ac4 vs. Ac5 comparison group could target four DEmiRNAs and then link to 17 DEmRNAs associated with 10 development-related signaling pathways (mTOR, Hedgehog, TGF-β, etc.) (Figure 5A). Furthermore, nine DEcircRNAs could target ace-miR-6001-y, further targeting 11 DEmRNAs relative to nine development-relevant signaling pathways (AMPK, Hippo, Insulin, etc.) (Figure 5B).

### 3.6 Regulatory networks relative to DEcircRNA/ace-miR-6001-y axis

**FIGURE 6:**
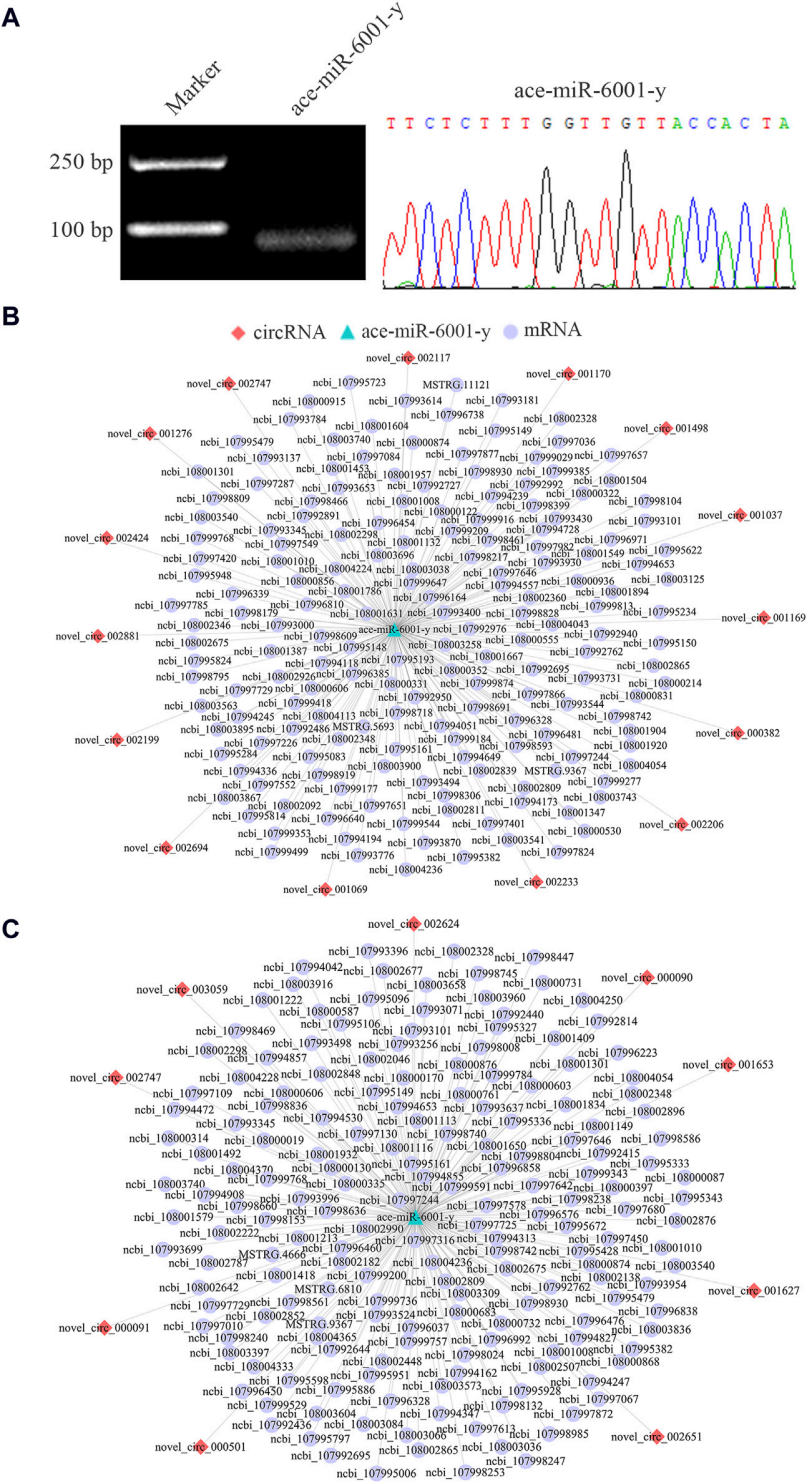
DEcircRNA/ace-miR-6001-y/DEmRNA networks in the *A. cerana* worker larval guts. **(A)**: Agarose gel electrophoresis for the stem-loop RT-PCR amplification product from ace-miR-6001-y and Sanger sequencing of target fragment. **(B, C)**: DEcircRNA/ace-miR-6001-y/DEmRNA networks in the Ac4 vs. Ac5 and Ac5 vs. Ac6 comparison group, respectively.

Since ace-miR-6001-y was a key target DEmiRNA present in the DEcircRNA-engaged ceRNA networks in both Ac4 vs. Ac5 and Ac5 vs. Ac6 comparison group, the molecular validation of ace-miR-6001-y was performed, followed by investigation of sub-networks relevant to DEcircRNA/ace-miR-6001-y axis, it’s detected that the fragment with expect size (approximately 59 bp) was amplified by stem-loop RT-PCR, and Sanger sequencing confirmed the sequence of ace-miR-6001-y (Figure 6A). In the Ac4 vs. Ac5 comparison group, 15 DEcircRNAs was detected to target ace-miR-6001-y, which further targeted 173 DEmRNAs (Figure 6B), involving 26 GO terms (signaling, transporter activity, synapse, etc.) and 142 KEGG pathways (lysosome, insulin secretion, TGF-β signaling pathway, etc.). Comparatively, nine DEcircRNAs in the Ac5 vs. Ac6 comparison group were observed to target ace-miR-6001-y, further targeting 177 DEmRNAs (Figure 6C) relevant to 26 functional terms such as localization and cell part and 199 pathways such as apoptosis and mTOR signaling pathway.

### 3.7 Molecular verification of back-splicing sites within four DEcircRNAs

**FIGURE 7:**
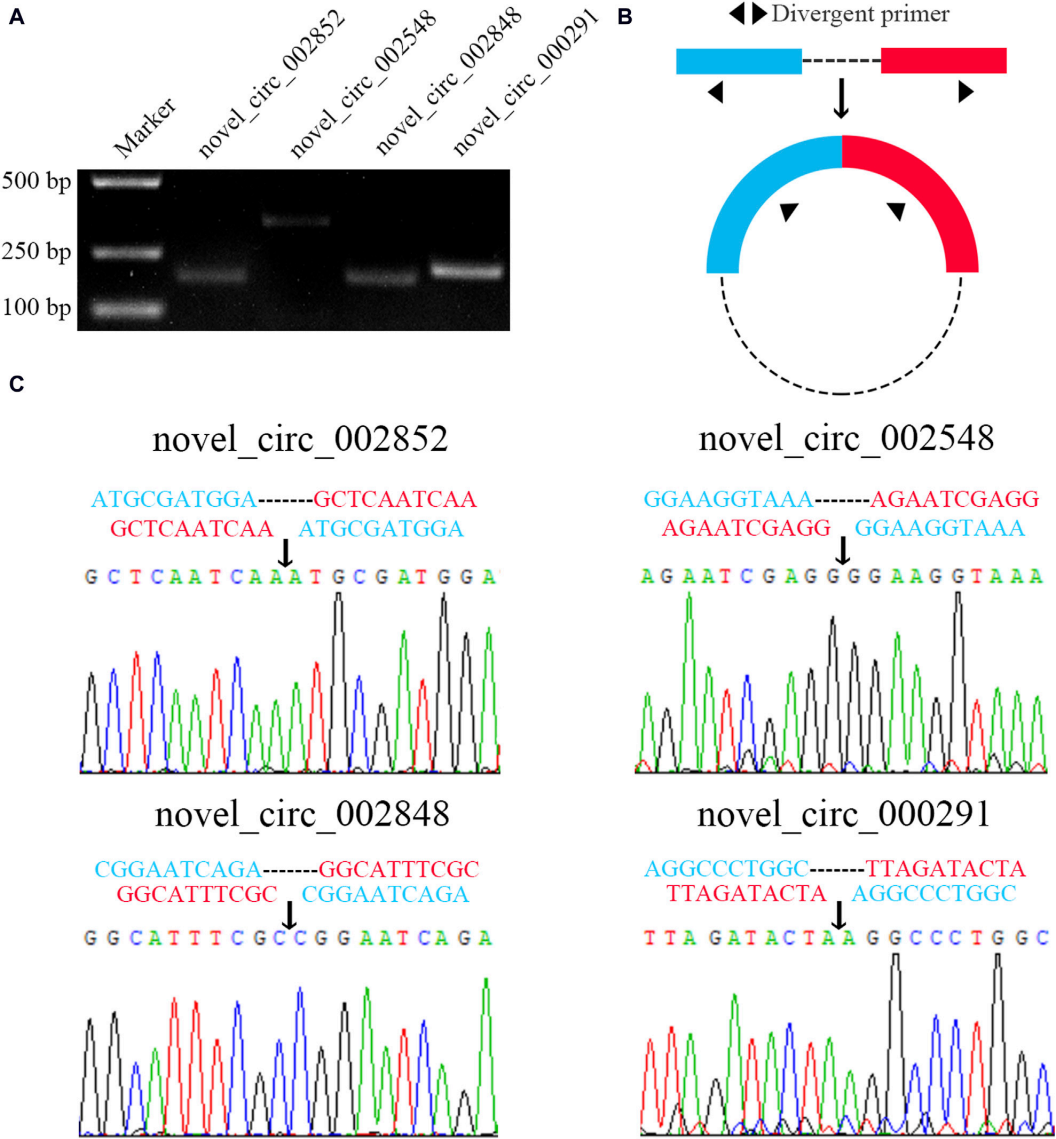
Molecular verification of back-splicing sites in four DEcircRNAs. **(A)**: Agarose gel electrophoresis for the PCR amplification products from four DEcircRNAs. **(B)**: Schematic diagram of divergent primers. **(C)**: Sanger sequencing of amplification products from four DEcircRNAs.

Four DEcircRNAs were randomly selected for PCR amplification, and the agarose gel electrophoresis demonstrated that the expected fragments (about 180, 349, 180, and 209 bp in size) could be amplified from four randomly selected DEcircRNAs (Figure 7A). In addition, the Sanger sequencing confirmed the authenticity of the back-splicing sites in these four DEcircRNAs (Figures 7B,C).

### 3.8 RT-qPCR detection of DEcircRNAs

**FIGURE 8:**
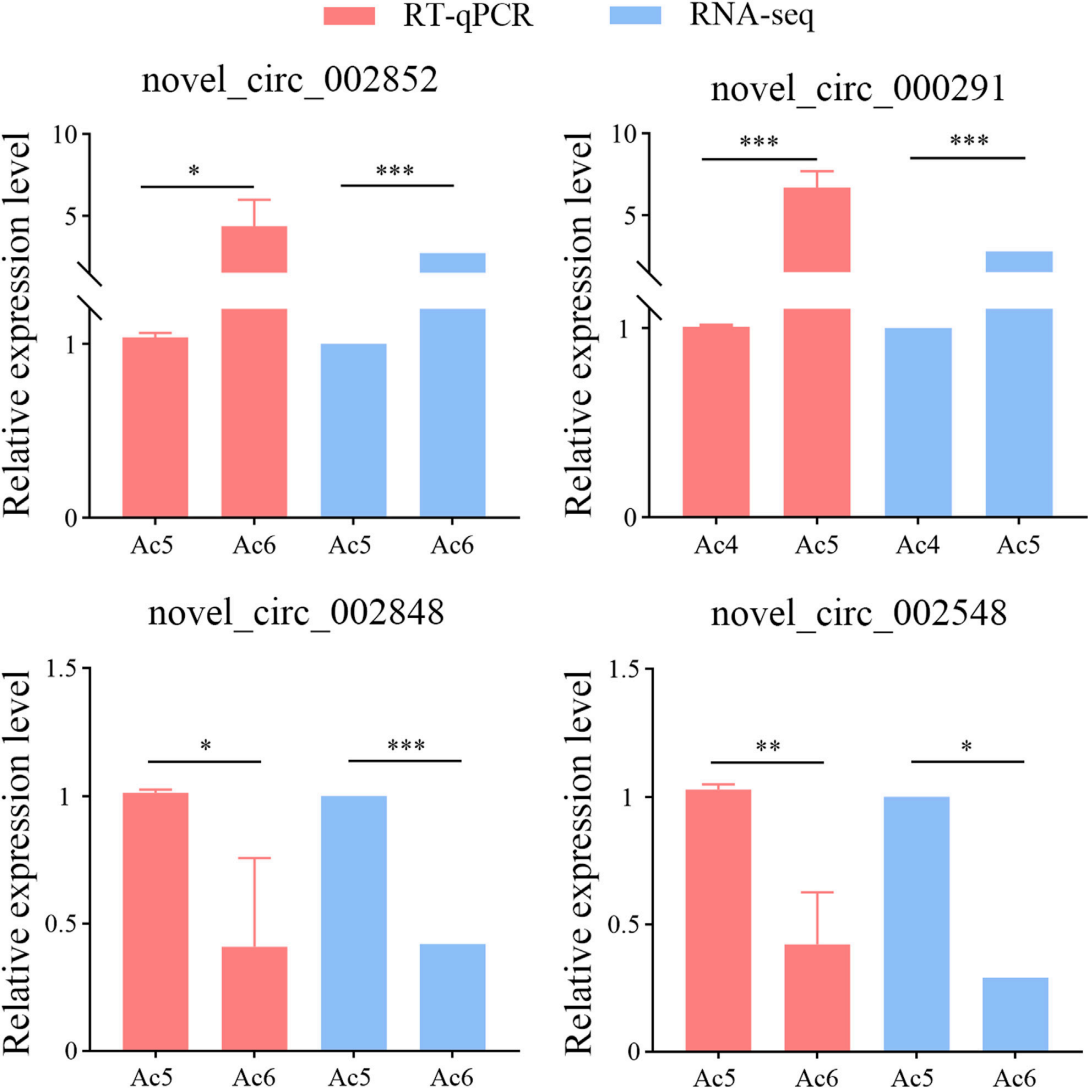
RT-qPCR assay of four DEcircRNAs. The qPCR data were presented as mean ± standard deviation (SD) and examined by two-sided Student’s t-test; ns, *p* > 0.05; *, *p* < 0.05; **, *p* < 0.01; ***, *p* < 0.001.

RT-qPCR was performed to prove the authenticity and reliability of sequencing data used in this work, the results indicated that the expression trends of these four DEcircRNAs were consistent with those in the transcriptome data, as shown in Figure 8.

### 3.9 Validation of the novel_circ_001627/ace-miR-6001-y and ace-miR-6001-y/ncbi_107995149 binding relationships

In insects, the *apterous* gene is engaed in the development of various tissues (Williams et al., 1993; Fernández-Fúnez et al., 1998). novel_circ_001627 (log_2_FC = −17.57, *p* = 0.03) was a significant DEcircRNA targeting ace-miR-6001-y, a key DEmiRNA within the ceRNA regulatory networks, which further targeted ncbi_107995149 (*apterous-like*). Hence, the novel_circ_001627/ace-miR-6001-y/*apterous-like* axis was chosen for verification of binding relationships.

**FIGURE 9:**
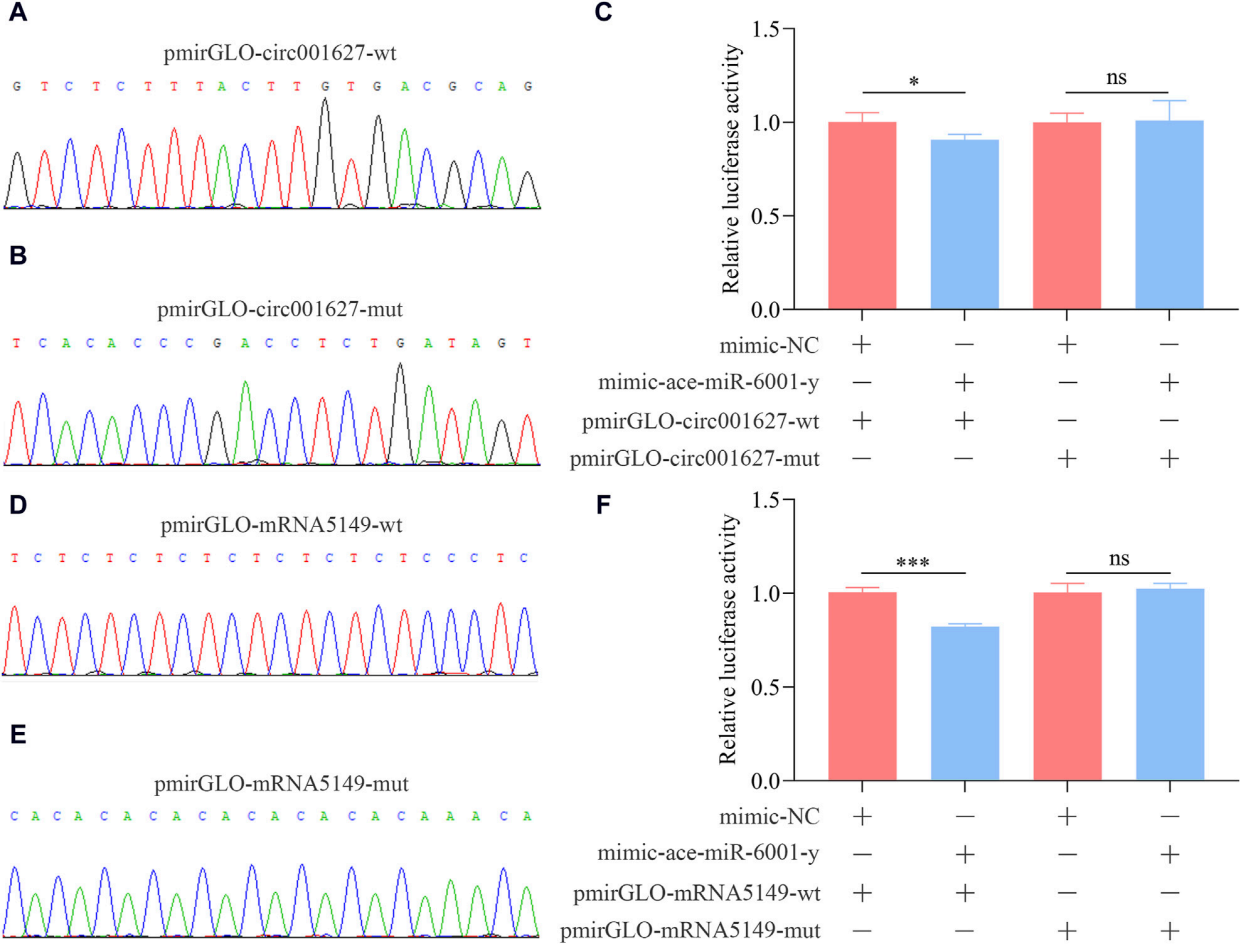
Confirmation of the binding relationships between novel_circ_001627 and ace-miR-6001-y and between ace-miR-6001-y and ncbi_107995149. **(A, D)**: Sanger sequencing of the amplified binding sites. **(B, E)**: Sanger sequencing of the mutated binding sites. **(C)**: Binding relationship between novel_circ_001627 and ace-miR-6001-y. **(F)**: Binding relationship between ncbi_107995149 and ace-miR-6001-y. The dual-luciferase assay data were presented as mean ± standard deviation (SD) and analyzed by two-sided Student’s t-test; ns, *p* > 0.05; *, *p* < 0.05; ***, *p* < 0.001.

As shown in Figures 9A,B,D,E, the recombinant plasmids pmir-GLO-circ001627-wt, pmirGLO-circ001627-mut, pmirGLO-mRNA5149-wt, and pmir-GLO-mRNA5149-mut were successfully constructed. The results of dual-luciferase assay suggested that the luciferase activities in the co-transfection group of mimic-ace-miR-6001-y and pmirGLO-circ001627-wt as well as the co-transfection group of mimic-ace-miR-6001-y and pmirGLO-mRNA5149-wt were significantly decreased compared to that in the corresponding control groups, whereas non-significant difference of the luciferase activity was observed between the co-transfection groups of mimic-ace-miR-6001-y and pmirGLO-circ001627-mut as well as the co-transfection group of mimic-ace-miR-6001-y and pmirGLO-mRNA5149-mut and the corresponding control groups. These results confirmed the binding relationships between novel_circ_001627 and ace-miR-6001-y as well as between ace-miR-6001-y and ncbi_107995149.

## 4 Discussion

Increasing studies have demonstrated that circRNAs were essential players in the developmental regulation of insects including *A. mellifera* (Krishnamoorthy and Kadener, 2021; Liu et al., 2022). Here, we detected 224 and 158 DEcircRNAs in the Ac4 vs. Ac5 and Ac5 vs. Ac6 comparison groups, respectively, including 42 shared ones (Figure 1). This indicated that the developmental process of *A. cerana* larval guts was accompanied with the differential expression of the overall circRNA profile, suggestive of the potential roles of these DEcircRNAs in the larval gut development. Findings from other groups have shown that not only circRNA but also other ncRNAs such as miRNAs, piRNAs, and lncRNAs were involved in the regulation of the development of insects (Maeda et al., 2018; Betting et al., 2021; Quesnelle et al., 2023). Previously, our team observed the differential expression of miRNAs, piRNAs, and lncRNAs in the developmental process of the *A. cerana* worker larval guts, and uncovered that DEmiRNAs, DEpiRNAs, and DElncRNAs were putative modulators in the larval gut development (Fan et al., 2023a; b; Long et al., 2022). In summary, these results suggested that various types of ncRNAs were likely to regulate the development of *A. cerana* worker larval guts through diverse manners such as ceRNA networks as both circRNAs and lncRNAs could interact with miRNAs (Tay et al., 2014; Zhang et al., 2019).

As yet, circRNAs have been verified to control the transcription of parental genes and further affect a series of biological processes like development and metabolism (Gao et al., 2023), immune response (Yin et al., 2021), and inhibiting cell proliferation (Li et al., 2018a). Li et al. (2018a) found that circITGA7 upregulated the transcription of its parental *ITGA*7 by suppressing *RREB*1 via the Ras pathway and inhibits the proliferation and metastasis of colorectal cancer (CRC) cells. Therefore, functional annotation and pathway analysis of corresponding parental genes have profound significance for understanding the role of circRNAs. For insects, the gut tissue is the major position for material metabolism (Zhang and Edgar, 2022). It’s found in this study that the parental genes of DEcircRNAs in the above-mentioned two comparison groups were engaged in 11 and seven metabolism-related pathways, respectively, such as glycerolipid metabolism, amino sugar and nucleotide sugar metabolism, and arginine biosynthesis (Figure 2E, see also Supplementary Figure S1). The results indicated that corresponding DEcircRNAs were potentially involved in modulating material metabolisms in the larval guts by regulating parental genes’ transcription.

The development of insect gut is a complex process modulated by different signaling pathways, such as Hippo, Wnt, and Hedgehog (Jacob and Lum, 2007; Shaw et al., 2010; Kuroda et al., 2012). Hippo signaling pathway plays a central part in controlling cell proliferation and fate as well as organ growth and regeneration (Ma et al., 2019). Here, novel_circ_001365 (log_2_FC = −17.68, *p* = 0.01) in the Ac4 vs. Ac5 comparison group was detected to downregulated and putatively regulated the transcription of the parental gene (ncbi_107997224) enriched in the Hippo signaling pathway. Also, two DEcircRNAs in the Ac5 vs. Ac6 comparison group, including novel_circ_001373 (log_2_FC = 3.18, *p* = 0.03) and novel_circ_002848 (log_2_FC = −1.25, *p* = 0.0003), were respectively observed to modulate the transcription of two parental genes (ncbi_107995510 and ncbi_108002348) involved in the Hippo signaling pathway. The results showed that novel_circ_001365, novel_circ_001373, and novel_circ_002848 may participate in the larval gut development via regulating the Hippo signaling pathway. Studies have shown that the Hedgehog signaling pathway is closely associated with the development of various organs or tissues, such as gastrointestinal tract, endocrine gland, skeletal development, and wing (van den Brink, 2007; Cohen, 2010; Ehlen et al., 2006; Vervoort, 2000). In this work, novel_circ_000076 (log_2_FC = −17.68, *p* = 0.01) and novel_circ_002942 (log_2_FC = −17.57, *p* = 0.03) in the Ac4 vs. Ac5 and Ac5 vs. Ac6 comparison group, was putatively modulated the transcription of ncbi_108004420 and ncbi_108002826, respectively, two genes engaged in the Hedgehog signaling pathway. This implied that novel_circ_000076 and novel_circ_002942 may regulate the development of larval gut in a Hedgehog signaling pathway-depending manner. Wnt signaling pathway plays a regulatory role of great importance in the development of organs in conjunction with the Hippo, Notch, and TGF-β signaling pathways (Sun and Wang, 2003; Barry and Camargo, 2013). Here, novel_circ_003093 (log_2_FC = −1.40, *p* = 5 × 10^−11^) was downregulated in the Ac4 vs. Ac5 comparison group and potentially regulated the transcription of a Wnt signaling pathway-relevant parental gene (ncbi_108003317), while novel_circ_002848 and novel_circ_002851 (log_2_FC = 1.49, *p* = 0.003) in the Ac5 vs. Ac6 comparison group respectively regulated the transcription of ncbi_108002348 and ncbi_108002373, two parental genes enriched in the Wnt signaling pathway, which suggested that these three DEcircRNAs were putative regulators in the gut development. Additionally, mTOR and AMPK are considered master regulators of cell metabolism and their activation is directly linked to the regulation of cellular metabolism, growth and survival (Garza-Lombó et al., 2018); similarly, mTOR controls Insulin signaling by regulating several downstream components (Yoon, 2017); TGF-β signaling pathway also plays an important role in cellular growth (Massagué and Sheppard, 2023). Here, novel_circ_000850 (log_2_FC = 17.77, *p* = 0.01) in the Ac4 vs. Ac5 comparison group and novel_circ_001609 (log_2_FC = 17.73, *p* = 0.01) in the Ac5 vs. Ac6 comparison group regulated the transcription of ncbi_107993192 and ncbi_107996586, respectively, involved in the AMPK signaling pathway. In the Ac4 vs. Ac5 comparison group, novel_circ_001175 (log_2_FC = 1.05, *p* = 0.02) and novel_circ_002087 (log_2_FC = −2.83, *p* = 0.0002) were respectively observed to modulate the transcription of ncbi_107994726 and ncbi_107998974, these two genes engaged in the Insulin signaling pathway. In the Ac5 vs. Ac6 comparison group, novel_circ_002848 (log_2_FC = −1.25, *p* = 0.0003) could regulate the transcription of ncbi_108002348, which could enrich in TGF-β signaling pathway. The above results suggested that these DEcircRNAs play a role in the development of the *A. cerana* larval guts by regulating the AMPK Insulin, TGF-β and mTOR signaling pathways. Among them, we found that in the Ac4 vs. Ac5 comparison group, novel_circ_002977 (log_2_FC = −3.19, *p* = 1 × 10^−11^) and novel_circ_002978 (log_2_FC = −2.04, *p* = 1 × 10^−6^) were both observed to modulate the transcription of ncbi_108002698, and the parental gene ncbi_108002698 could involve in the AMPK, mTOR, and Insulin signaling pathway. This implied that novel_circ_002977 and novel_circ_002978 may regulate the development of larval gut through the combined action of mTOR/AMPK signaling pathway and Insulin signaling pathway. This also provides an important idea for our future research, next, we may explore the role of novel_circ_002977 and novel_circ_002978 in the development of the *A. cerana* worker larval guts in more detail. Together, these results demonstrated that corresponding DEcircRNAs were likely to modulate the Hippo, Hedgehog, Wnt, mTOR, AMPK, Insulin, and TGF-β signaling pathways by regulating the transcription of parental genes, further affecting the developmental process of the *A. cerana* worker larval guts.

The insect gut is a critical immune organ responsible for detection and defense against invading pathogens or parasites (Ratcliffe, 1985; Vilmos and Kurucz, 1998). In the present study, the parental genes of DEcircRNAs in the Ac4 vs. Ac5 comparison group were associated with four cellular immune pathways (lysosome, endocytosis, phagosome, and melanogenesis) and four humoral immune pathways (MAPK signaling pathway, MAPK signaling pathway-fly, cAMP signaling pathway, and Ras signaling pathway) (Figure 2C), whereas the parental genes of DEcircRNAs in the Ac5 vs. Ac6 comparison group were relative to nine immune pathways such as apoptosis and JAK-STAT signaling pathway (Supplementary Figure S1). In addition, the parental genes of three DEcircRNAs (novel_circ_002852, novel_circ_002853, novel_circ_002687) shared by these two comparison groups were found to regulate the parental genes engaged cAMP signaling pathway, and MAPK signaling pathway. The results were indicative of the involvement of corresponding DEcircRNAs in modulating the cellular and humoral immune in the larval gut during the developmental process.

Accumulating evidence have shown that various types of ncRNAs, such as circRNAs and lncRNAs, are able to regulate diverse activities of insects like development and immunity through ceRNA networks using miRNAs as a bridge (Gao et al., 2023; Ren et al., 2023). CircRNAs, mRNAs, and any other RNAs that share common miRNA response elements (MREs) can competitively bind with miRNAs, acting as an RNA sponge to block and inhibit miRNAs from binding to their target sites (Tay et al., 2014). Studies found that ceRNA regulatory networks are widely present in the growth and development of various insects, such as *A. albopictus* (Liu et al., 2023), *Tetranychus cinnabarinus* (Feng et al., 2022), *B. mori* (Yin et al., 2021). Here, we noticed that 19 DEcircRNAs (novel_circ_002117, novel_circ_001037, and novel_circ_002747, etc.) in the Ac4 vs. Ac5 comparison group could simultaneously target four DEmiRNAs (ace-novel-m0032-5p, ace-miR-6001-y, ace-miR-6001-x, and ace-miR-980-y), further targeting 14 DEmRNAs (ncbi_107993074, ncbi_108001413, and ncbi_108003740, etc.) which engaged in Hippo, Wnt, Hedgehog, mTOR, AMPK, Insulin, and TGF-β signaling pathways (Figure 5A); equally, nine DEcircRNAs such as novel_circ_000090 and novel_circ_002624 in the Ac5 vs. Ac6 comparison group could bind with ace-miR-6001-y and than targeted 11 DEmRNAs which also involved in Hippo, Wnt, Hedgehog, etc. seven signaling pathways (Figure 5B). The above results indicated that circRNAs in *A. cerana* worker larval guts development by regulating these seven signaling pathways through the ceRNA regulatory networks. Moreover, it also suggested that circRNAs can regulate the same pathway in different regulatory ways (regulating the transcription of parental genes and ceRNA mechanism). Insect Notch signaling plays remarkably diverse roles in development to regulate cell fate determination, organ growth and tissue patterning (Chen Y. et al., 2023). And the Notch signaling plays essential roles during ovary development (Shcherbata et al., 2004), leg growth (Cordoba and Estella, 2020), and wing development (Blair, 2007) in *Drosophila melanogaster*. In this work, we noticed that 15 DEcircRNAs in the Ac4 vs. Ac5 comparison group targeted ace-miR-6001-y further targeting ncbi_107998104 which enriched in Notch signaling pathway. Unlike the way of regulating the transcription of parental genes, the Notch signaling pathway is only regulated by the ceRNA mechanism, so we speculated the Notch signaling pathway may be a special pathway that can be regulated by the ceRNA mechanism during the development of *A. cerana* worker larval guts. In addition, these target DEmRNAs were annotated to seven (eight) cellular immune pathways and five (eight) humoral immune pathways, including melanogenesis, lysosome, Jak-STAT signaling pathway, and cAMP signaling pathway (Figure 4). This confirmed that the corresponding DEcircRNAs were involved in the modulation of immunity during gut development in *A. cerana* larvae.

Collins et al. (2017) isolated and validated Bte-miR-6001-5p and Bte-miR-6001-3p, and found that Bte-miR-6001-5p and Bte-miR-6001-3p were more highly expressed in queen-than in worker-destined late-instar larvae in *Bombus terrestris*. Shan et al. (2023) indicated that ame-miR-6001-3p was a key factor in circRNA-mediated ceRNA regulatory networks in the *A. mellifera* brain tissue which exposed to fluvalinate. Furthermore, ame-miR-6001-5p was found to play an essential role in ecdysone secretion and caste differentiation in *A. mellifera* (Shi et al., 2015; Ashby et al., 2016). Recently, Fan et al. (2023b) analyzed the expression pattern, regulatory network, and putative role of miRNAs through the small RNA-seq data from *A. cerana* worker larvae, and found that ace-miR-6001-y was upregulated in both Ac4 vs. Ac5 and Ac5 vs. Ac6 comparison groups and adjusted some great importance signaling pathways (Wnt, Hippo, and Jak-STAT signaling pathways, etc.). More recently, Fan et al. (2023a) found that 54 and 116 DElncRNAs in the Ac4 vs. Ac5 and Ac5 vs. Ac6 comparison groups, respectively, acted as ceRNAs to competitively target ame-miR-6001-y and regulated the development of *A. cerana* worker larval guts. Here, 15 DEcircRNAs in the Ac4 vs. Ac5 comparison group, such as novel_circ_000382, novel_circ_001276, and novel_circ_002424, were observed to target ace-miR-6001-y, further targeting 173 DEmRNA, involving eight (Hippo, Wnt, and AMPK signaling pathway, etc.) and 11 (necroptosis, melanogenesis, and lysosome, etc.) pathways relevant to development and immunity, respectively (Figure 6B). Additionally, ace-miR-6001-y was observed to be targeted by nine DEcircRNAs (novel_circ_000090, novel_circ_001627, and novel_circ_002651, etc.) in the Ac5 vs. Ac6 comparison group and further targeted 177 DEmRNAs (Figure 6C). These targets were engaged in nine development-related pathways including Hippo signaling pathway and Hedgehog signaling pathway as well as 16 immune-related pathways including MAPK signaling pathway and apoptosis. These results together demonstrated that corresponding DEcircRNAs were likely to absorb ace-miR-6001-y and further affect the development and immunity of the *A. cerana* larval guts. In recent years, *adaptors* have been shown to be associated with the development of ventral and dorsal compartments of the wing imaginal disc in *Drosophila* (Williams et al., 1993; Fernández-Fúnez et al., 1998). Here, we randomly selected novel_circ_001627/ace-miR-6001-y/*adaptors-like* (ncbi_107995149) axis for dual-luciferase reporter assay. The results verified the authenticity of the bonding relationship (Figure 9) and indicated that novel_circ_001627 and ace-miR-6001-y were possibly involved in the developmental process of *A. cerana*. This laied the groundwork for a more in-depth exploration in the future.

**FIGURE 10:**
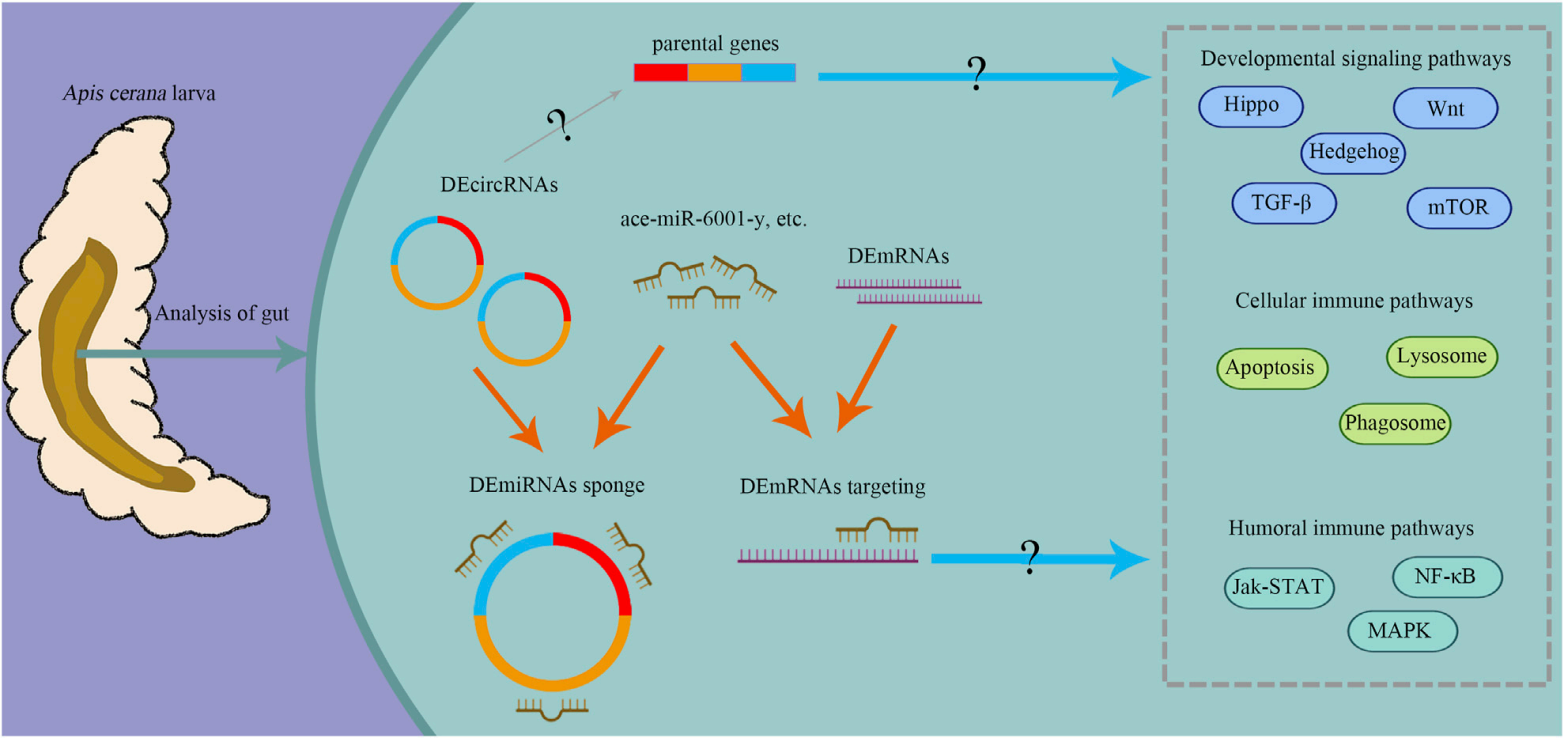
A hypothetical working model of circRNA regulation of gut development in *A. cerana* worker larva. The orange arrows indicate the competitive binding of DEmiRNAs between DEcircRNAs and DEmRNAs; the blue arrows indicate that DEcircRNAs may regulate important pathways through the action of their parental genes and ceRNA mechanism, the gray arrow indicate that DEcircRNAs may participate in the development and immune by modulating corresponding parental genes, “?” indicate that regulation is based on the results from bioinformatic analyses in this current work. This diagram was created with MedPeer website (www.medpeer.cn).

To summarize, circRNAs were dynamically expressed in the development of *A. cerana* workers larval gut. And during the developmental process, DEcircRNAs could be involved in regulation through their parental genes and as ceRNAs to compete with mRNAs to bind the same miRNAs (Figure 10). Some essential pathways (Hedgehog, Wnt, and Hippo signaling pathway) and the DEcircRNA/ace-miR-6001-y/DEmRNA axis have been found to act during the development of *A. cerana* workers larval guts. In a word, all these results reveal the role of cricRNA regulation during development. Dual-luciferase reporter assay verified the novel_circ_001627/ace-miR-6001-y/*adaptors-like* axis’ target relationships and provides a basis for corresponding circRNAs research.

## Data Availability

The datasets presented in this study can be found in online repositories. The names of the repository/repositories and accession number(s) can be found in the article/Supplementary Material.
