# Supplementary figures and images for: Unraveling the modulatory manner and function of circRNAs in the Asian honey bee larval guts
Source: Front Cell Dev Biol. 2024 Jul 9;12:1391717. doi: 10.3389/fcell.2024.1391717 (PMC11263028; doi:10.3389/fcell.2024.1391717)

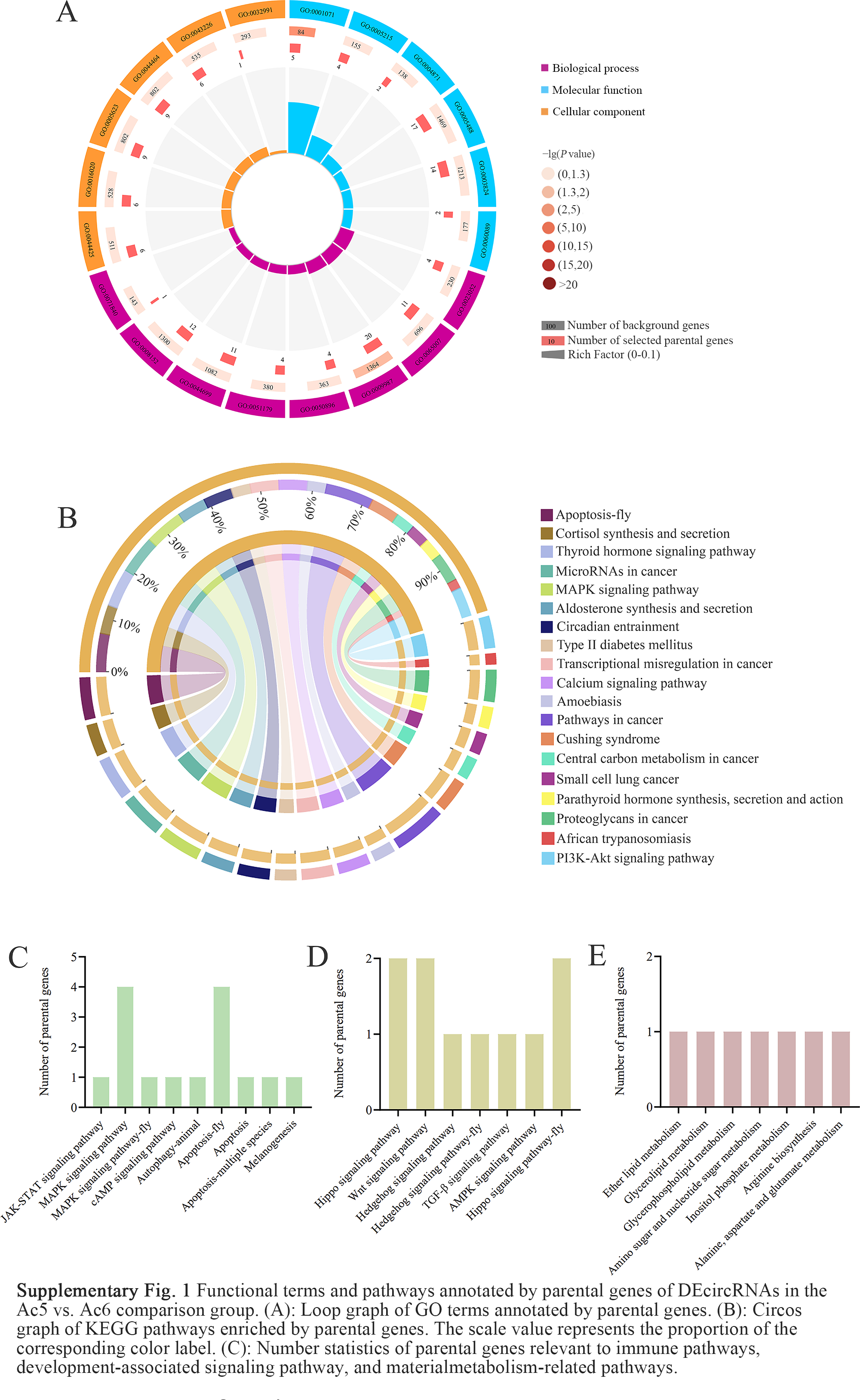

Supplement: Supplementary file 4 [file Image1.TIF]
